# Tolerating bad health research: the continuing scandal

**DOI:** 10.1186/s13063-022-06415-5

**Published:** 2022-06-02

**Authors:** Stefania Pirosca, Frances Shiely, Mike Clarke, Shaun Treweek

**Affiliations:** 1 Health Services Research Unit, University of Aberdeen, Foresterhill, Aberdeen, AB25 2ZD UK; 2 Trials Research and Methodologies Unit, HRB Clinical Research Facility, University College Cork, Cork, Ireland; 3 School of Public Health, University College Cork, Cork, Ireland; 4 Northern Ireland Methodology Hub, Queen’s University Belfast, Belfast, UK

**Keywords:** Randomised trials, Research waste, Risk of bias, Statisticians, Methodologists

## Abstract

**Background:**

At the 2015 REWARD/EQUATOR conference on research waste, the late Doug Altman revealed that his only regret about his 1994 *BMJ* paper ‘The scandal of poor medical research’ was that he used the word ‘poor’ rather than ‘bad’. But how much research is bad? And what would improve things?

**Main text:**

We focus on randomised trials and look at scale, participants and cost. We randomly selected up to two quantitative intervention reviews published by all clinical Cochrane Review Groups between May 2020 and April 2021. Data including the risk of bias, number of participants, intervention type and country were extracted for all trials included in selected reviews. High risk of bias trials was classed as bad. The cost of high risk of bias trials was estimated using published estimates of trial cost per participant.

We identified 96 reviews authored by 546 reviewers from 49 clinical Cochrane Review Groups that included 1659 trials done in 84 countries. Of the 1640 trials providing risk of bias information, 1013 (62%) were high risk of bias (bad), 494 (30%) unclear and 133 (8%) low risk of bias. Bad trials were spread across all clinical areas and all countries. Well over 220,000 participants (or 56% of all participants) were in bad trials. The low estimate of the cost of bad trials was £726 million; our high estimate was over £8 billion.

We have five recommendations: trials should be neither funded (1) nor given ethical approval (2) unless they have a statistician and methodologist; trialists should use a risk of bias tool at design (3); more statisticians and methodologists should be trained and supported (4); there should be more funding into applied methodology research and infrastructure (5).

**Conclusions:**

Most randomised trials are bad and most trial participants will be in one. The research community has tolerated this for decades. This has to stop: we need to put rigour and methodology where it belongs — at the centre of our science.

**Supplementary Information:**

The online version contains supplementary material available at 10.1186/s13063-022-06415-5.

## Background

At the 2015 REWARD/EQUATOR conference on research waste, the late Doug Altman revealed that his only regret about his 1994 *BMJ* paper ‘The scandal of poor medical research’ [1] was that he used the word ‘poor’ rather than ‘bad’. Towards the end of his life, Doug had considered writing a sequel with a title that included not only ‘bad’ but ‘continuing’ [2].

That ‘continuing’ is needed should worry all of us. Ben Van Calster and colleagues have recently highlighted the paradox that science consistently undervalues methodology that would underpin good research [3]. The COVID-19 pandemic has generated an astonishing amount of research and some of it has transformed the way the virus is managed and treated. But we expect that much COVID-19 research will be bad because much of health research in general is bad [3]. This was true in 1994 and it remains true in 2021 because how research is done allows it to be so. Research waste seems to be baked-in to the system.

In this commentary, we do not intend to list specific examples of research waste. Rather, we want to talk about scale, participants and money and then finish with five recommendations. All of the latter will look familiar — Doug Altman and others [3–8] have suggested them many times — but we hope our numbers on scale, participants and money will lend the recommendations an urgency they have always deserved but never had.

## So, how much research is bad?

That research waste is common is not in doubt [3–8] but we wanted to put a number on something more specific: how much is bad research that is not just wasteful but which we could have done without and lost little or nothing? Rather than trying to tackle all of health research, we have chosen to focus on randomised trials because that is the field we know best and, in addition, they play a central role in decisions regarding the treatments that are offered to patients.

With this in mind, we aimed to estimate the proportion of trials that are bad, how many participants were involved and how much money was spent on them.

## Selecting a cohort of trials

We used systematic reviews as our starting point because these bodies of trial evidence often underpin clinical practice through guideline recommendations and policy. We specifically chose Cochrane systematic reviews because they are standardised, high-quality systematic reviews. We were only interested in recent reviews because these represent the most up-to-date bodies of evidence.

Moreover, Cochrane reviews record the review authors’ judgements about the risk of bias of included trials, in other words, they assess the extent to which the trial’s findings can be believed [9]. We consider that to be a measure of how good or bad a trial is. Cochrane has three categories of overall risk of bias: high, uncertain and low. We considered a high risk of bias trial to be bad, a low risk of bias trial to be good and an uncertain risk of bias trial to be exactly that, uncertain. We did not attempt to look at which type (or ‘domain’) of bias drove the overall assessment. We share the view given in the Cochrane Handbook (Chapter 8) [9] that the overall risk of bias is the least favourable assessment across the domains of bias. If one domain is high risk, then the overall assessment is high risk. No domain is more or less important than any other and if there is a high risk of bias in even just one domain, this calls into question the validity of the trial’s findings.

We used the list randomiser at random.org to randomly select two reviews published between May 2020 and April 2021 from each of the 53 clinical Cochrane Review Groups. To be included, a review had to consider intervention effects rather than being a qualitative review or a review of reviews. We then extracted basic information (our full dataset is at https://osf.io/dv6cw/?view_only=0becaacc45884754b09fd1f54db0c495) about every included trial in each review, including the overall risk of bias assessment. Our aim was to make no judgements about the risk of bias ourselves but to take what the review authors had provided. We did not contact the review or trial authors for additional information. Extracted data were put into Excel spreadsheets, one for each Cochrane Review Group.

## Analysis

To answer our question about the proportion of bad trials and how many participants were in them, we used simple counts across reviews and trials. Counts across spreadsheets were done using R and our code is at https://osf.io/dv6cw/?view_only=0becaacc45884754b09fd1f54db0c495. To estimate how much money might have been spent on the trials, we used three estimates of the cost-per-participant to give a range of possible values for total spend:

1. Estimate 1: An estimate of the cost-per-participant for the UK’s National Institute for Health Research Health Technology Assessment (NIHR HTA) Programme trials of 2987 GBP. This was calculated based on a median cost per NIHR HTA trial of 1,433,978 GBP for 2011–2016 [10] and a median final recruitment target for NIHR HTA trials of 480 for 2004–2016 [11].
2. Estimate 2: The median cost-per-participant of 41,413 USD found for pivotal clinical benefit trials supporting the US approval of new therapeutic agents, 2015–2017 [12].
3. Estimate 3: The 2012 average cost-per-participant for UK trials of 9758 EUR found by Europe Economics [13].

These estimates were all converted into GBP using https://www.currency-converter.org.uk to get the exchange rate on 1st January in the latest year of trials covered by the estimate (i.e. 2017 for E2 and 2012 for E3). These were then all converted to 2021 GBP on 11 August 2021 using https://www.inflationtool.com, making E1 £3,256, E2 £35,918 and E3 £9,382. We acknowledge that these are unlikely to be exact for any given trial in our sample, but they were intended to give ballpark average figures to promote discussion.

## Scale, participants and money

### Scale

We extracted data for 1659 randomised trials spread across 96 reviews from 49 of the 53 clinical Cochrane Review Groups. The remaining four Review Groups published no eligible reviews in our time period. The 96 included reviews involved 546 review authors. Trials in 84 countries, as well as 193 multinational trials, are included. Risk of bias information was not available for 19 trials, meaning our risk of bias sample is 1640 trials. Almost all reviews (94) exclusively used Cochrane’s original risk of bias tool (see Supplementary File 1) rather than the new Risk of Bias tool (version 2.0) [14]. Cochrane RoB 1.0 has six domains of bias (sequence generation; allocation concealment; blinding of participants, personnel and outcome assessors; incomplete outcome data; selective outcome reporting; other sources of bias), while RoB 2.0 has five domains (randomisation process; assignment and adherence to intervention; incomplete outcome data; outcome measurement; selective reporting). Where the old tool was used, we used review authors’ assessment of the overall risk of bias. For the two reviews that used Risk of Bias 2, we did not make individual risk of bias judgements for domains, but we did take a view on the overall risk of bias if the review authors did not do this. We did this by looking across the individual domains and making a choice of high, uncertain or low overall risk of bias based on the number of individual domains falling into each category. This was a judgement; we did not use a hard-and-fast rule. We had to do this for 40 trials.

**Fig. 1:**
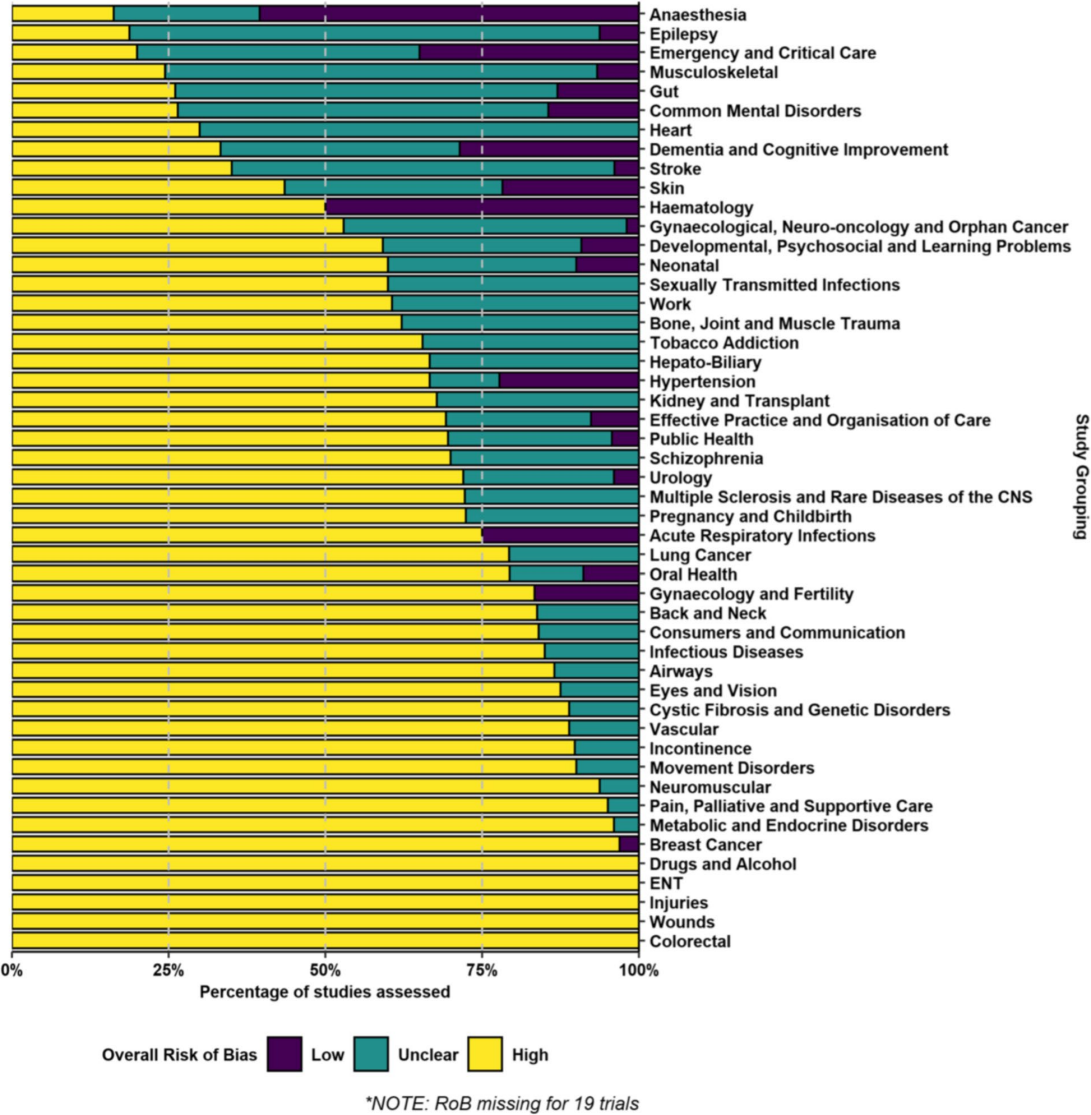
Risk of bias for included trials in randomly selected systematic reviews published between May 2020 and April 2021 by 49 Cochrane Review Groups

The majority of trials (1013, or 62%) were high risk of bias (Table 1). These trials were spread across all 49 Cochrane Review Groups and over half of the Groups (28, or 57%) had zero low risk of bias trials included in the reviews we randomly selected. The clinical area covered by the Anaesthesia Review Group had the highest proportion of low risk of bias trials at 60% but this group included 19 trials with no risk of bias information (see Fig. 1).

**Table 1:** Risk of bias and number of participants for the included trials

	High	Unclear	Low	No RoB
**Number of trials** **(total = 1659; % based on 1640 trials with risk of bias information)**	1013 (62%)	494 (30%)	133 (8%)	19
**Number of participants** **(total = 398,410)**	222,850 (55.93%)	127,290 (31.95%)	47,138 (11.83%)	1132 (0.28%)

Some of the 84 countries in our sample contributed very few trials but Table 2 shows risk of bias data for the 17 countries that contributed 20 or more trials, as well as for multinational trials. The percentage of a country’s trials that were judged as low risk of bias reached double figures for multinational trials (23%) and five individual countries: Australia (10%), France (13%), India (10%), Japan (10%) and the UK (11%). The full country breakdown is given in Supplementary File 2.

**Table 2:** Risk of bias for trials done in countries contributing 20 or more trials

Country	Number of trials	High	Unclear	Low
Multinational	193	100 (52%)	48 (25%)	45 (23%)
Australia	41	27 (66%)	10 (24%)	4 (10%)
Brazil	27	16 (59%)	10 (37%)	1 (4%)
Canada	40	26 (65%)	12 (30%)	2 (5%)
China	87	56 (64%)	23 (26%)	7 (8%)
France	31	11 (35%)	16 (52%)	4 (13%)
Germany	48	40 (83%)	7 (15%)	1 (2%)
India	48	35 (73%)	6 (13%)	5 (10%)
Iran	49	30 (61%)	18 (37%)	0 (0%)
Italy	47	30 (64%)	17 (36%)	0 (0%)
Japan	42	22 (52%)	16 (38%)	4 (10%)
Korea	32	18 (56%)	11 (34%)	1 (3%)
Netherlands	22	17 (77%)	5 (23%)	0 (0%)
Spain	29	25 (86%)	2 (7%)	1 (3%)
Sweden	26	16 (62%)	7 (27%)	2 (8%)
Turkey	32	18 (56%)	9 (28%)	2 (6%)
UK	114	63 (55%)	38 (33%)	12 (11%)
USA	280	168 (60%)	98 (35%)	12 (4%)

### Participants

The 1659 included trials involved a total of 398,410 participants. The majority of these (222,850, or 56%) were in high risk of bias trials (Table 1).

### Money

Table 3 shows estimates for the amount of money spent on trials in each of the three risk of bias categories.

**Table 3:** The estimated cost of high, uncertain and low risk of bias trials

Risk of bias	Number of trials	Participants	E1 costs (£)	E2 costs (£)	E3 costs (£)
High	1013	222,850	725,599,600	8,004,326,300	2,090,778,700
Uncertain	494	127,290	414,456,240	4,572,002,220	1,194,234,780
Low	133	47,138	153,481,328	1,693,102,684	442,248,716

Using our low estimate for cost-per-participant (estimate 1 from NIHR HTA trials), we get an estimated spend of £726 million on high risk of bias trials. Our high estimate (estimate 2 from USA drug approval trials) gives an equivalent figure of over £8 billion. Based on an annual spend of £76 million for the UK’s NIHR HTA programme [15], the first figure, our lowest estimate, would be sufficient to fund the programme for almost a decade, while the second figure would fund it for over a century.

While looking at scale, participants and money, we made a few other secondary observations. To avoid distracting attention from our main points, we present these observations in Supplementary File 3.

## Discussion

Bad trials — ones where we have little confidence in the results — are not just common, they represent the majority of trials across all clinical areas in all countries. Over half of all trial participants will be in one. Our estimates suggest that the money spent on these bad trials would fund the UK’s largest public funder of trials for anything between a decade and a century. It is a wide range but either way, it is a lot of money. Had our random selection produced a different set of reviews, or we had assessed all those published in the last 1, 5, 10 or 20 years, we have no reason to believe that the headline result would have been different. Put simply, most randomised trials are bad.

Despite this, we think our measure of bad is actually conservative because we have only considered the risk of bias. We have not attempted to judge whether trials asked important research questions, whether they involved the right participants and whether their outcomes were important to decision-makers such as patients and health professionals nor have we attempted to comment on the many other decisions that affect the usefulness of a trial [16, 17]. In short, the picture our numbers paint is undoubtedly gloomy, but the reality is probably worse.

## Five recommendations for change

Plenty of ideas have been suggested about what must change [1, 3–8], but we propose just five here because the scale of the problem is so great that providing focus might avoid being overwhelmed into inaction. We think these five recommendations, if implemented, would reduce the number of bad trials and could do so quite quickly.

### Recommendation 1: do not fund a trial unless the trial team contains methodological and statistical expertise

Doing trials is a team sport. These teams need experienced methodologists and statisticians. We do not know how many trials fail to involve experienced methodologists and statisticians but we expect it to be a high proportion given the easily avoidable design errors seen in so many trials. It is hard to imagine doing, say, bowel surgery without involving people who have been trained in, and know how to do, bowel surgery. Sadly, the same does not seem to be true for trial design and statistical analysis of trial data. Our colleague Darren Dahly, a trial statistician, neatly captured the problem in a series of ironic tweets sent at the end of 2020:

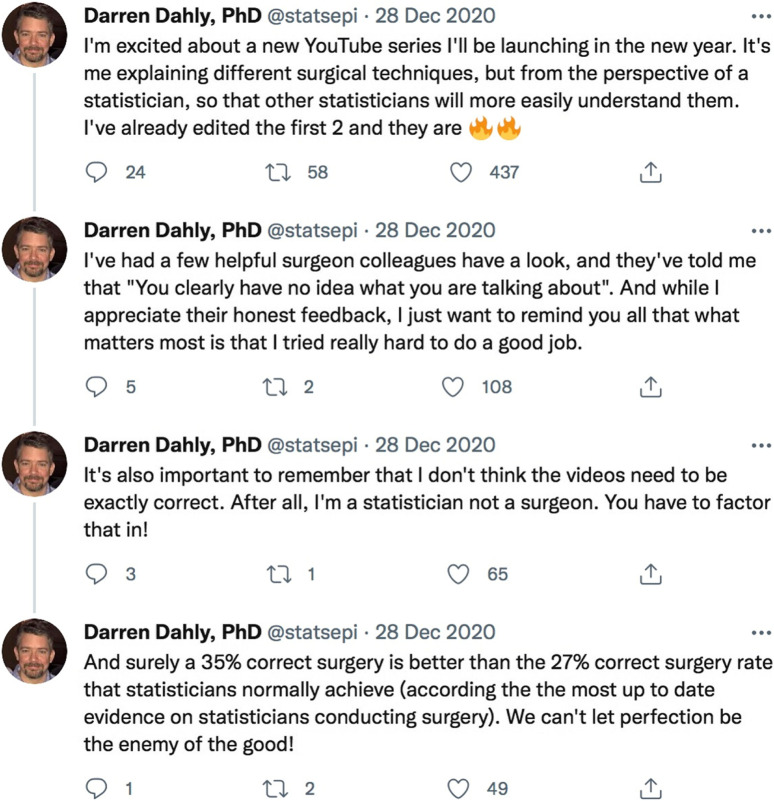



These raise a smile but make a very serious point: we would not tolerate statisticians doing surgery so why do we tolerate the reverse? Clearly, this is not about surgeons, it is about not having the expertise needed to do the job properly.

### Recommendation 2: do not give ethical approval for a trial unless the trial team contains methodological and statistical expertise

As for recommendation 1, but for ethical approval. All trials need ethical approval and the use of poor methods should be seen as an ethical concern [3]. No patient or member of the public should be in a bad trial and ethical committees, like funders, have a duty to stop this happening. Ethics committees should always consider whether there is adequate methodological and statistical expertise within the trial team. Indeed, we think public and patient contributors on ethics committees should routinely ask the question ‘Who is the statistician and who is the methodologist?’ and if the answer is unsatisfactory, ethical approval is not awarded until a name can be put against these roles.

### Recommendation 3: use a risk of bias tool at trial design

This is the simplest of our recommendations. Risk of bias tools were developed to support the interpretation of trial results in systematic reviews. However, as Yordanov and colleagues wrote in 2015 [5], by then the horse has bolted and nothing can be changed. They considered 142 high risk of bias trials and found the four most common methodological problems to be exclusion of patients from analysis (50 trials, 35%), lack of blinding with a patient-reported outcome (27 trials, 19%), lack of blinding when comparing a non-drug treatment to nothing (23 trials,16%) and poor methods to deal with missing data (22 trials, 15%). They judged the first and last of these to be easy to fix at the design stage, while the two blinding problems were more difficult but not impossible to deal with. Sadly, trial teams themselves had not addressed any of these problems.

Applying a risk of bias tool at the trial design phase, having the methodological and statistical expertise to correctly interpret the results and then making any necessary changes to the trial, would help to avoid some of the problems highlighted by others [3–8] in the past and which we have found to be very common.

Applying a risk of bias tool at the trial design phase, having the methodological and statistical expertise to correctly interpret the results and then making any necessary changes to the trial, would help to avoid some of the problems we and others [3–8] highlight. Funders could ask to see the completed risk of bias tool, as could ethics committees. No trial should be high risk of bias.

### Recommendation 4: train and support more methodologists and statisticians

Recommendations 1, 2 and 3 all lead to a need for more methodologists and statisticians. This has a cost but it would probably be much less than the money wasted on bad trials. See recommendation 5.

### Recommendation 5: put more money into applied methodology research and supporting infrastructure

Methodology research currently runs mostly on love not money. This seems odd when over 60% of trials are so methodologically flawed we cannot believe their results and we are uncertain whether we should believe the results of another 30%.

In 2015, David Moher and Doug Altman proposed that 0.1% of funders’ and publishers’ budgets could be set aside for initiatives to reduce waste and improve the quality, and thus value, of research publications [6]. That was for publications but the same could be done for trials, although we would suggest a figure closer to 10% of funders’ budgets. All organisations that fund trials should also be funding applied work to improve trial methodology, including supporting the training of more methodologists and statisticians. There should also be funding mechanisms to ensure methodology knowledge is effectively disseminated and implemented. Dissemination is a particular problem and the UK’s only dedicated methodology funder, the Medical Research Council-NIHR ‘Better Methods, Better Research’ Panel, acknowledges this in its Programme Aims [18].

Implementing these five recommendations will require effort and investment but doing nothing is not an option that anyone should accept. We have shown that 220,850 people had been enrolled in trials judged to be so methodologically flawed that we can have little confidence in their results. A further 127,290 people had joined trials where it is unclear whether we should believe the results. These numbers represent 88% of all trial participants in our sample. This is a betrayal of those participants’ hopes, goodwill and time. Even our lowest cost-per-participant estimate would suggest that more than £1billion was spent on these bad and possibly bad trials.

The question for everyone associated with designing, funding and approving trials is how many good trials never happen because bad ones are done instead? The cost of this research waste is not only financial. Randomised trials have the potential to improve health and wellbeing, change lives for the better and support economies through healthier populations. But poor evidence leads to poor decisions [19]. Society will only see the potential benefits of randomised trials if these studies are good, and, at the moment, most are not.

In this study, we have concentrated on risk of bias. What makes our results particularly troubling is that the changes needed to move a trial from high risk of bias to low risk of bias are often simple and cheap. However, this is also positive in relation to changing what will happen in the future. For example, Yordanov and colleagues estimated that easy methodological adjustments at the design stage would have made important improvements to 42% (95% confidence interval = 36 to 49%) of trials with risk of bias concerns [5]. Their explanation for these adjustments not being made in the trials was a lack of input from methodologists and statisticians at the trial planning stage combined with insufficient knowledge of research methods among the trial teams. If we were to ask a statistician to operate on a patient, we would rightly fear for the patient: proposing that a trial is designed and run without research methods expertise should induce the same fear.

In 2009, Iain Chalmers and Paul Glasziou estimated that 85% of research spending is wasted due to, among other things, poor design and incomplete reporting [7]. Over a decade later, our estimate is that 88% of trial spending is wasted. Without addressing the fundamental problem of trials being done by people ill-equipped to do them, a similar study a decade from now will once again find that the majority of trials across all clinical areas in all countries are bad.

Our work, and that of others before us [1, 3–8], makes clear that a large amount of the money we put into trials globally is being wasted. Some of that money should be repurposed to fund our five recommendations. This may well lead to fewer trials overall but it would generate more good trials and mean that a greater proportion of trial data is of the high quality needed to support and improve patient and public health.

## Conclusion

That so much research, and so many trials, is and are bad is indeed a scandal. That it continues decades after others highlighted the problem is a bigger scandal. Even the tiny slice of global research featured in our study describes trials that involved hundreds of thousands of people and cost hundreds of millions of pounds, but which led to little or no useful information.

The COVID-19 pandemic has been a time for many things, including reflection. As many countries start to look to what can be learnt, all of us connected with trials should put rigour and methodology where it belongs — at the centre of our science. We think our five recommendations are a good place to start.

To quote Doug Altman ‘We need less research, better research, and research done for the right reasons’ [1]. Quite so.

## Supplementary Information

**Additional file 1.** The Cochrane Collaboration’s ‘old’ tool for assessing risk of bias.

**Additional file 2.** Risk of bias data for all countries in our sample.

**Additional file 3.** Additional observations.

## Data Availability

All our data are available at https://osf.io/dv6cw/?view_only=0becaacc45884754b09fd1f54db0c495.

## Abbreviations

BMJ: British Medical Journal
COVID-19: Coronavirus disease 2019
E1: Estimate 1
E2: Estimate 2
E3: Estimate 3
EUR: Euro
GBP: British Pound Sterling
HTA: Health Technology Assessment
NIHR: National Institute for Health Research
UK: United Kingdom
USA: United States of America
USD: United States Dollar
