# Supplementary material for: Tolerating bad health research: the continuing scandal
Source: Trials. 2022 Jun 2;23:458. doi: 10.1186/s13063-022-06415-5 (PMC9161194; doi:10.1186/s13063-022-06415-5)

**Supplementary File 2**

While estimating the proportion of trials that are bad, how many participants were involved and how much money was spent were our main questions, we did make a few other secondary observations. We provide them here because some readers might find them interesting.

**Factors that might affect the risk of bias**

We found that trials with multiple recruitment sites were twice as likely to be low risk of bias (12%) than trials with a single recruitment site
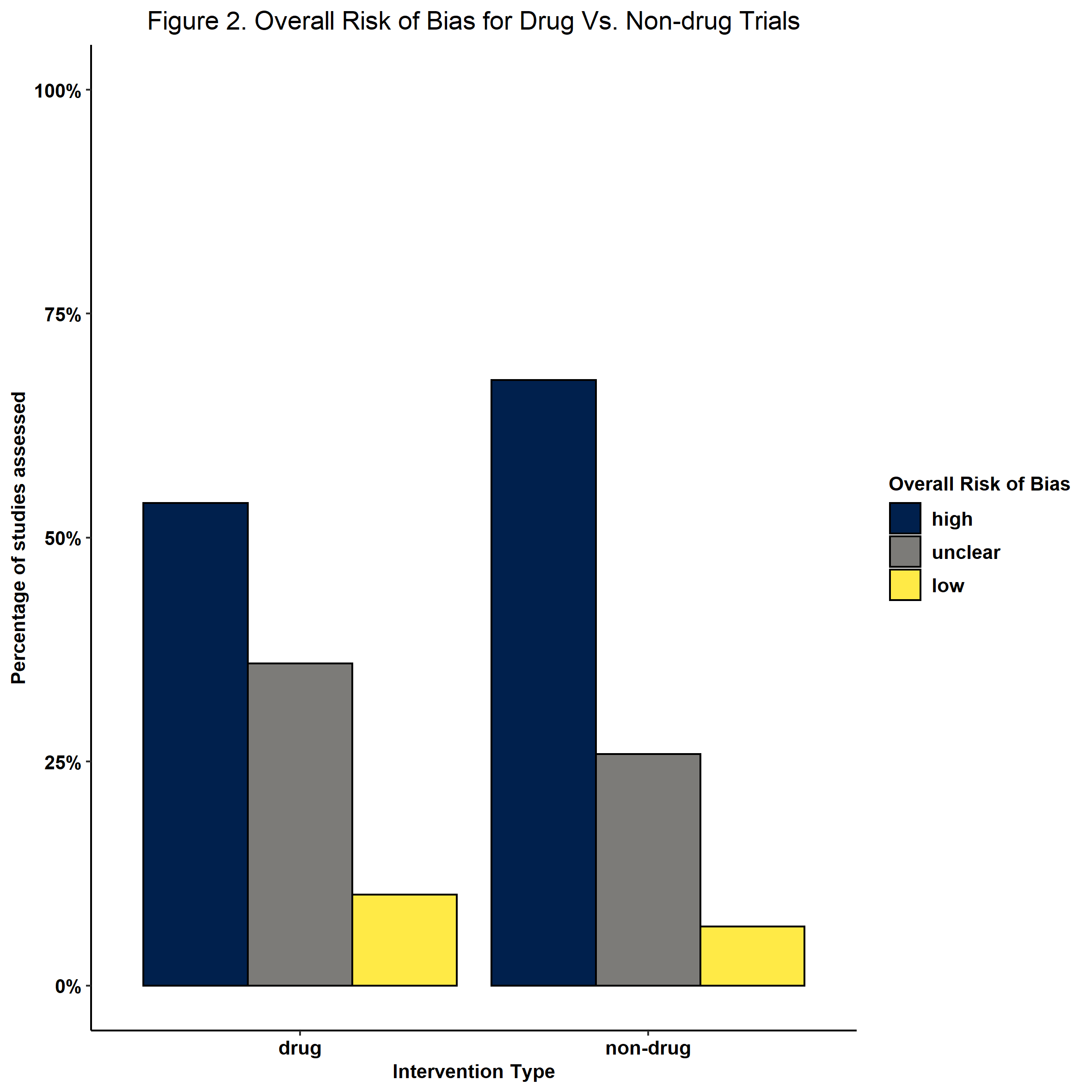
 (6%). We also found that trials with multiple recruitment sites had a slightly lower proportion of high risk of bias trials (59%) than trials with just one recruitment site (66%).

We also found that the proportion of drug trials that were low risk of bias was higher than for than non-drug trials (10% compared to 6%, respectively) and that the proportion of high risk of bias drug trials was lower than for non-drug trials (54% compared to 66%, respectively) (Figure S1).

We are uncertain as to why this might be the case. Perhaps trials teams running trials outside their own centre, and those doing drug trials, may have better access to methodological and statistical support. This is pure speculation though and would need further investigation.

**Signs of hope?**

Doug Altman published his paper in 1994 and we looked at how risk of bias in trials has evolved since then. The picture is broadly similar from 1994 to 2021: there are far more high risk of bias trials than low risk of bias trials (see Figure S2). However, if we are actively looking for a positive sign we could say that the proportion of low risk of bias trials started, possibly, to increase slowly from around the mid-2000s. Roughly speaking we could say that the proportion of low risk of bias trials was comfortably below 10% until the mid-2000s and was somewhere between 10% and 20% thereafter.

Cochrane introduced its risk of bias tool in 2006 and perhaps this has been a factor although we have no way of knowing. Even if there is a link, the impact of the uncoordinated use of the risk of bias tool at trial design is modest. Most trials remain high risk of bias. If we are kind and say the proportion of low risk of bias trials has increased from about 10% in 2005 to about 20% in 2021, then at that rate of improvement we could expect all our trials to be low risk of bias trials by the middle of the 22nd century.

**Figure S2**: the proportion of trials in our sample at high, uncertain and low risk of bias between 1994 and 2021.


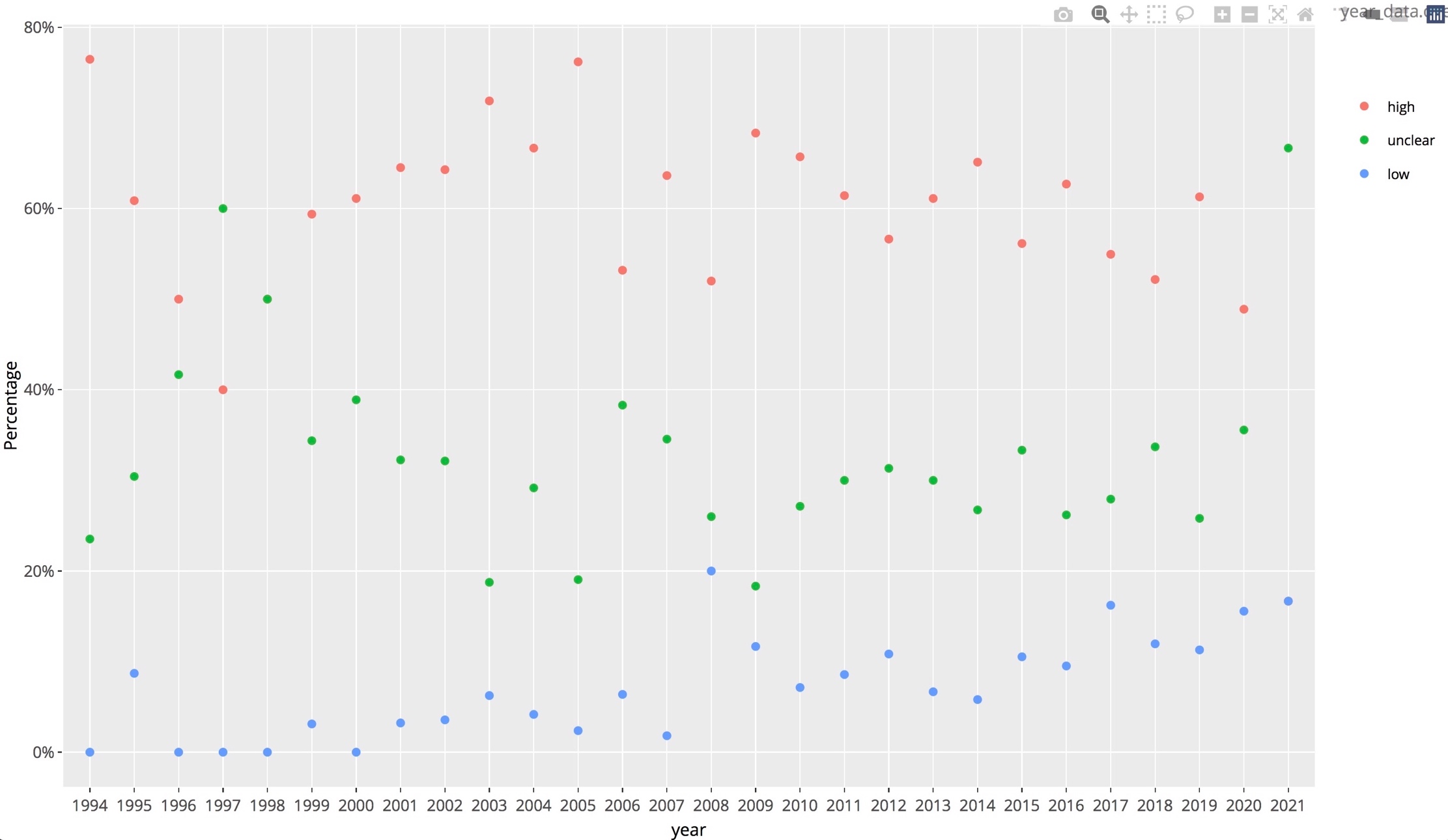

Supplement: Supplementary file 3 — Additional file 3. Additional observations. [file 13063_2022_6415_MOESM3_ESM.docx]
